# Tuberculosis Caused by *Mycobacterium africanum*, United States, 2004–2013

**DOI:** 10.3201/eid2203.151505

**Published:** 2016-03

**Authors:** Aditya Sharma, Emily Bloss, Charles M. Heilig, Eleanor S. Click

**Affiliations:** Author affiliation: Centers for Disease Control and Prevention, Atlanta, Georgia, USA

**Keywords:** Mycobacterium africanum, bacteria, tuberculosis and other mycobacteria, surveillance, genotype, United States

## Abstract

*Mycobacterium africanum* is endemic to West Africa and causes tuberculosis (TB). We reviewed reported cases of TB in the United States during 2004–2013 that had lineage assigned by genotype (spoligotype and mycobacterial interspersed repetitive unit variable number tandem repeats). *M. africanum* caused 315 (0.4%) of 73,290 TB cases with lineage assigned by genotype. TB caused by *M. africanum* was associated more with persons from West Africa (adjusted odds ratio [aOR] 253.8, 95% CI 59.9–1,076.1) and US-born black persons (aOR 5.7, 95% CI 1.2–25.9) than with US-born white persons. TB caused by *M. africanum* did not show differences in clinical characteristics when compared with TB caused by *M. tuberculosis*. Clustered cases defined as >2 cases in a county with identical 24-locus mycobacterial interspersed repetitive unit genotypes, were less likely for *M. africanum* (aOR 0.1, 95% CI 0.1–0.4), which suggests that *M. africanum* is not commonly transmitted in the United States.

## Abstract 2

Routine reporting of TB caused by this organism does not appear warranted at this time.

Tuberculosis (TB) is an infectious disease caused by a group of highly-related organisms comprising the *Mycobacterium tuberculosis* complex (MTBC), which includes *M. tuberculosis*, *M. africanum*, and *M. bovis*. Although all members of MTBC might cause disease in humans, *M. tuberculosis* and *M. africanum* are the primary cause of disease in humans globally, whereas *M. bovis* primarily causes disease in cattle (*1*,*2*). Like *M. tuberculosis, M. africanum* is spread by aerosol transmission (*3*).

Phylogenetic analysis has suggested there are 7 major lineages of MTBC, designated L1–L7 (*4*,*5*). *M africanum* was traditionally identified by using biochemical methods. However, molecular methods have shown that *M. africanum* is composed of 2 distinct lineages: L5 (also known in other nomenclature systems as *M. africanum* West African 1 [MAF1], West African lineage I), which is genetically part of *M. tuberculosis* sensu stricto, and L6 (also known as *M. africanum* West African 2 [MAF2], West African lineage II), which is genetically more similar to *M. bovis* (*4*–*9*).

Among lineages that primarily infect humans, *M. africanum* lineages are considered phylogenetically more ancient relative to the modern lineages of *M. tuberculosis* (Euro-American, East African Indian, East Asian). *M. africanum* has been described as endemic to equatorial Africa, with specimens isolated from countries such as Nigeria, Côte d’Ivoire, Benin, Senegal, Cameroon, Burkina Faso, The Gambia, Sierra Leone, and Uganda (*8*,*10*–*21*). *M. africanum* has also been isolated from patients with TB in countries in Europe (*22*–*25*), Brazil (*26*), and the United States (*27*). It is likely that TB caused by *M. africanum* in non-African countries is secondary to human migration from disease-endemic areas in equatorial Africa (*25*).

Several studies have explored whether there are clinical differences between TB caused by *M. africanum* and TB caused by *M. tuberculosis*. These studies demonstrated variable findings with regard to associations of *M. africanum* with HIV status and findings on chest radiography (*8*,*28*–*30*). Contacts of persons with TB caused by *M. africanum* appeared to have a lower rate of progression to active TB compared with contacts of persons with TB caused by *M. tuberculosis*, and a lower rate of genotype clustering has been described for *M. africanum* than for *M. tuberculosis* in relatively small studies from West Africa (*14**,**29*).

Although bacterial strains causing TB from all over the world can be found among cases of TB in the United States, analysis of routinely collected genotyping data for 2005–2009 showed that 179 (0.5%) of 36,458 TB cases reported nationally were caused by *M. africanum* (*31*). We sought to further expand knowledge of *M. africanum* in the United States by reviewing all cases of TB reported nationally during 2004–2013. The objectives of this study were to ascertain the proportion of TB cases caused by *M. africanum* in the United States; compare clinical and epidemiologic characteristics between *M. africanum* and *M. tuberculosis*; and determine the extent to which *M. africanum* strains in the United States might be related by transmission on the basis of genotype clustering.

## Methods

Genotype data from the Centers for Disease Control and Prevention (CDC; Atlanta, GA, USA) National TB Genotyping Service for 2004 through 2013 were linked to routine demographic and clinical data from all culture-confirmed cases in the CDC National TB Surveillance System from all 50 US states and the District of Columbia (*32*). As described previously (*33*), phylogenetic lineage (*M. africanum* and *M. tuberculosis*) for TB cases was assigned on the basis of spoligotype by using a set of rules correlating spoligotype to lineages defined by large sequence polymorphisms; for cases that did not meet a full rule for assignment on the basis of spoligotype, 12-locus mycobacterial interspersed repetitive unit variable number tandem repeats (MIRU-VNTRs) was used in addition to spoligotype to assign lineage. Cases reported during 2004–2008 only had 12-locus MIRU-VNTR data available, and cases reported during 2009–2013 had 24-locus MIRU-VNTR data available. To identify cases that could be caused by ongoing transmission in the United States, clusters of cases were defined as >2 cases with the same spoligotype and 24-locus MIRU-VNTR pattern in a given county. Cases that were caused by organisms other than *M. africanum* or *M. tuberculosis* were excluded from analysis.

All analyses were conducted by using R statistical software version 3.0.1 (R Core Group, Vienna, Austria). Statistical test results were considered significant at p<0.05. We examined patient attributes, genotype clustering, clinical characteristics (e.g., disease site), and social risk factors (e.g., homelessness) associated with *M. africanum* and *M. tuberculosis*. Odd ratios (ORs) and 95% CIs were calculated. Differences in proportions of cases were detected by using Fisher exact and Pearson χ^2^ tests.

Factors identified as statistically significant by bivariable analysis at p<0.05 were entered into a multivariable logistic regression model to assess whether these factors were independently associated with *M. africanum* and *M. tuberculosis*. Tolerance <0.10 was used to detect co-linearity, and the likelihood ratio test was used to test for interaction. To address collinearity between race/ethnicity and origin of birth, variables for race/ethnicity, country of origin, and West African origin were combined into a single variable and included in selection of the multivariable regression model. West African origin was defined as having been born in any of the following countries in West Africa: Nigeria, Liberia, Sierra Leone, Guinea, The Gambia, Ghana, Mali, Senegal, Côte d’Ivoire, Togo, Cameroon, Mauritania, Niger, and Guinea-Bissau.

### Ethics Statement

Data for this study were collected as part of routine TB surveillance by CDC. Thus, this study was not considered research involving human subjects, and institutional review board approval was not required.

## Results

**Figure 1:**
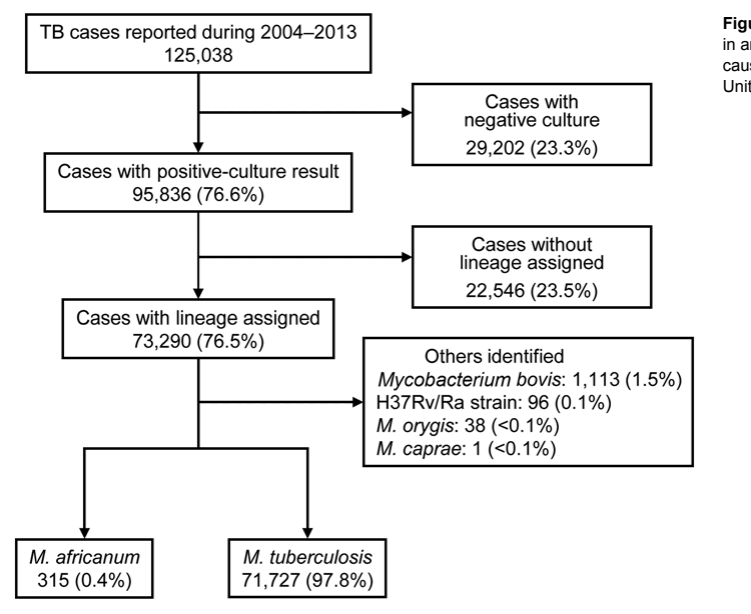
Selection of cases included in analysis of tuberculosis (TB) caused by *Mycobacterium africanum*, United States, 2004–2013.

A total of 125,038 cases were reported to the National TB Surveillance System during 2004–2013 (Figure 1). Of these cases, 95,836 (76.6%) had a culture result positive for MTBC. Of cases with positive culture results, 73,290 (76.5%) had available lineage identification on the basis of genotype data. Of the cases for which lineage identification was available, the causative agent was determined to be *M. africanum* for 315 (0.4%) and *M. tuberculosis* for 71,727 (97.9%) cases: 1,248 (1.7%) cases had an isolated organism other than *M. africanum* or *M. tuberculosis* and were excluded from further analysis (Figure 1).

*M. africanum* was assigned as the causative agent of TB for isolates with a genotype-assigned lineage of L5 or L6. All isolates designated as *M. africanum* met the conventional spoligotype rule of the absence of spacers 8, 9, and 39 or the absence of spacers 7–9 and 39 (*7*). *M. tuberculosis* was assigned as the causative agent of TB for isolates with a genotype-assigned lineage of L1, L2, L3, L4, or L7.

Of the 315 case-patients with TB caused by *M. africanum*, 155 (49.2%) had the L5 lineage and 160 (50.8%) had the L6 lineage. Case-patients with the L5 lineage were most commonly born in Nigeria (n = 76), Liberia (n = 12), and Ghana (n = 12), and case-patients with the L6 lineage were most commonly born in Liberia (n = 27), Sierra Leone (n = 22), Guinea (n = 17), and The Gambia (n = 16).

Among case-patients with *M. africanum* as the causative agent of TB, 276 (87.6%) had country of birth other than the United States (Technical Appendix Table 1). Of the 276 foreign-born persons with *M. africanum*, most (254, 92.0%) persons were born in countries in West Africa, such as Nigeria (79, 31.1%), Liberia (39, 15.4%), and Sierra Leone (24, 9.4%).

**Figure 2:**
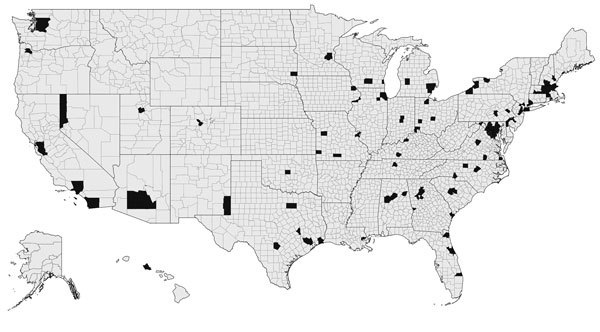
Counties in the United States with *Mycobacterium africanum* infections identified among tuberculosis (TB) cases (black) reported during 2004–2013.

Among all US states, 35 reported >1 case of TB caused by *M. africanum* (Figure 2). States that reported more than >10 cases of *M. africanum* TB during the study were New York (n = 77), Maryland (n = 41), Texas (n = 26), Virginia (n = 19), Georgia (n = 15), and California (n = 14). Across the United States, many reported cases of *M. africanum* TB appeared to be near major metropolitan areas, such as Atlanta, Georgia; Chicago, Illinois; Detroit, Michigan; Houston, Texas; Los Angeles, California; New York, New York; and Washington, DC.

**Figure 3:**
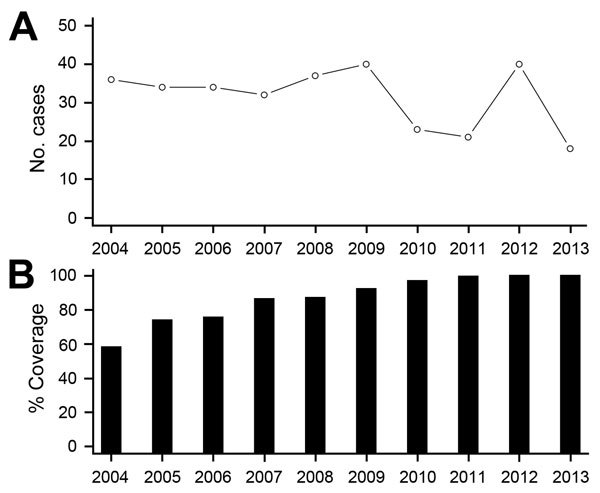
A) Annual number of reported *Mycobacterium africanum* tuberculosis cases and B) corresponding percentage of national genotype surveillance coverage, United States, 2004–2013.

The annual number of reported TB cases identified with *M. africanum* in the United States during 2004–2013 ranged from 18 to 40 (median 34 annual cases) (Figure 3). During this period, the proportion of *Mycobacterium* spp. TB isolates from persons born in West Africa with culture-confirmed TB that were genotyped ranged from 68.0% to 97.1%, which was comparable with the overall proportion of culture-confirmed TB cases that were genotyped nationally.

On the basis of the genotype cluster definition of >2 cases in the same county with identical spoligotype and 24-locus MIRU-VNTR patterns, only 1 cluster of *M. africanum* cases was identified during 2009–2013. The cluster consisted of 2 case-patients with the L5 lineage: 1 foreign-born person and 1 US-born person.

Among 315 cases of *M. africanum* TB, 183 distinct genotypes were identified (spoligotype and 12-locus MIRU-VNTR available for cases reported during 2004–2013; Technical Appendix Table 2). Of these 183 genotypes, 139 (76.0%) were found in a single case only; the remaining 44 (24.0%) caused 176 cases. Among 141 *M. africanum* cases reported during 2009–2013 with spoligotype and 24-locus MIRU-VNTR data available, 123 distinct genotypes were identified (Technical Appendix Table 3). Of these 123 genotypes, 113 (91.9%) were found in isolates from 1 case only, and 10 (8.1%) were found in >1 case.

Bivariable analysis showed that *M. africanum* and *M. tuberculosis* TB cases had major differences for several characteristics (Technical Appendix Table 1). When compared with *M. tuberculosis* TB cases, *M. africanum* TB cases had higher odds of being in foreign-born persons (odds ratio [OR] 4.8, 95% CI 3.4–6.7), being in non-Hispanic black or multiracial non-Hispanic persons (OR 27.0, 95% CI 17.1–42.5), originating from countries in West Africa (OR 318.4, 95% CI 239.0–424.2), being in persons positive for HIV (OR 2.8, 95% CI 2.0–3.7), and being in persons with only extrapulmonary disease (OR 1.8, 95% CI 1.4–2.4) or in persons with pulmonary and extrapulmonary disease (OR 1.6, 95% CI 1.1–2.2).

*M. africanum* TB cases had lower odds than *M. tuberculosis* TB cases of being in a cluster (defined by spoligotype and 24-locus MIRU) of cases (OR 0.1, 95% CI 0.1–0.2), being in persons >65 years of age (OR 0.2, 95% CI 0.1–0.5), being in persons with an abnormal chest radiographic result and cavitation (OR 0.6, 95% CI 0.5–0.9) and in persons without cavitation (OR 0.5, 95% CI 0.4–0.7), being in a resident of a correctional facility (OR 0.2, 95% CI 0.0–0.6), being in a homeless person (OR 0.4, 95% CI 0.2–0.8), being in persons reporting excessive drug (OR 0.2, 95% CI 0.1–0.5) or alcohol use (OR 0.2, 95% CI 0.1–0.4), and being in persons who died during treatment (OR 0.3, 95% CI 0.2–0.7). Among foreign-born persons, *M. africanum* TB cases had lower odds than *M. tuberculosis* TB cases of being in persons who had been in the United States for >5 years before reporting TB (OR 0.3, 95% CI 0.3–0.5).

Multivariable analysis restricted to cases reported during 2009–2013 that had 24-locus MIRU-VNTR data available showed that foreign-born West African origin (OR 253.8, 95% CI 59.9–1076.1) and US-born non−Hispanic black race (OR 5.7, 95% CI 1.2–25.9) were independently associated with TB caused by *M. africanum* but not with TB caused by *M. tuberculosis* (Table). Clustered cases (OR 0.1, 95% CI 0.1–0.4) had lower adjusted odds of TB caused by *M. africanum* than TB caused by *M. tuberculosis*. Other risk factors were not independently associated with *M. africanum* versus *M. tuberculosis*. No significant interaction terms were identified.

**Table:** Multivariable analysis of risk factors associated with tuberculosis caused by *Mycobacterium africanum* and *M. tuberculosis*, United States, 2009–2013

Risk factor	Adjusted OR (Wald 95% CI)
Combined race/ethnicity and origin	
Foreign born, non−West African	0.4 (0.1–1.9)
Foreign born, West African	253.8 (59.9–1076.1)
US born, non-Hispanic black	5.7 (1.2–25.9)
US born, Hispanic or other non-Hispanic race	1.1 (0.2–8.0)
US born, non-Hispanic white	Referent
Clustered case	
Yes	0.1 (0.1–0.4)
No	Referent
Age, y	
0–14	Referent
15–24	1.0 (0.3–3.5)
25–44	0.8 (0.2–2.5)
45–64	0.6 (0.2–2.0)
>65	0.3 (0.1–1.3)
Sex	
F	0.9 (0.6–1.4)
M	Referent
Reported HIV status	
Negative	Referent
Positive	0.9 (0.5–1.4)
Unknown/not determined	1.7 (0.9–3.3)
Primary disease site	
Pulmonary	Referent
Extrapulmonary	1.9 (1.0–3.6)
Pulmonary and extrapulmonary	1.1 (0.6–2.3)
Chest radiography finding	
Abnormal, cavitary	2.1 (1.0–4.5)
Abnormal, noncavitary	0.9 (0.5–1.7)
Normal	Referent
Homeless in year before diagnosis	
Yes	1.0 (0.3–3.0)
No	Referent
Resident of correctional facility in year before diagnosis	
Yes	0.6 (0.1–4.8)
No	Referent
Any drug use	
Yes	0.4 (0.1–1.9)
No	Referent
Excessive alcohol use	
Yes	0.8 (0.3–2.4)
No	Referent
Reason therapy stopped	
Completed treatment	Referent
Died during treatment	0.2 (0.1–1.6)
Other reason	1.3 (0.5–3.2)

To control for possible host differences in larger analysis, we conducted a subanalysis of cases among foreign-born persons from West Africa. In this subanalysis, clustering was the only significant variable at the bivariable level, and *M. africanum* TB cases had lower odds of being in a cluster of cases than *M. tuberculosis* TB cases (OR 0.1, 95% CI 0.1–0.9). Among foreign-born persons with West African origin, we found no significant differences in clinical characteristics (e.g., HIV status, cavitary disease, sputum smear results) between TB cases caused by *M. africanum* versus those caused by *M. tuberculosis*. *M. africanum* TB cases with L5 and L6 lineages had similar proportions of HIV positivity (18.1% vs. 17.5%; p = 0.9) and cavitary disease by chest radiography (25.4% vs. 42.5%; p = 0.051). We found no significant differences in clinical characteristics or social risk factors for TB caused by L5 or L6 lineages.

## Discussion

This study used nationally reported data on TB cases linked to genotype data to describe the epidemiology of *M. africanum* in the United States. The findings from this analysis indicate that *M. africanum* is a rare cause of TB in the United States and represents 315 (0.4%) of 73,290 cases with available genotype data reported during 2004–2013. Most cases were identified in large metropolitan areas throughout the United States. Although *M. africanum* is an infrequent cause of TB, most states reported >1 case of TB caused by *M. africanum* during the study period, which suggested that *M. africanum* is broadly distributed.

In this study, TB caused by *M. africanum* was more likely to occur in foreign-born West Africans and US-born non-Hispanic blacks and less likely in foreign-born persons originating from countries not in West Africa. These associations suggest that the epidemiology of *M. africanum* in the United States is driven primarily by migration of persons from West Africa. We also identified cases of *M. africanum* in US-born persons, primarily in non-Hispanic blacks. This finding suggests that transmission of *M. africanum* might occur in the United States, but the possibility of acquisition of TB during travel (e.g., to West Africa) cannot be excluded because travel history was not available in national surveillance data. In an initial report of 5 *M. africanum* cases in the United States, several case-patients did not report a history of travel to West Africa (*34*).

The low proportion of TB cases attributed to *M. africanum* suggests decreased transmissibility in the United States. Reasons for decreased transmission of *M. africanum* are unknown but could include decreased infectiousness or decreased progression to disease compared with *M. tuberculosis*, as was previously reported (*8*).

Our findings support the observation that *M. africanum* is highly restricted to West Africa, where it has been estimated to cause up to 50% of all TB cases, although the reason for this restriction remains unclear (*8*). A recent study from Ghana reported an association between *M. africanum* and patient ethnicity, which suggests specificity of host−pathogen interaction could be 1 factor in limiting the spread of *M. africanum* to West Africa (*35*).

Most *M. africanum* TB cases were not part of genotype clusters, which suggested that transmission of *M. africanum* in the United States is not common. *M. africanum* TB cases were less likely to be associated with genotype clustering than *M. tuberculosis* TB cases by analyses of all cases reported in the United States and in a subanalysis of persons born in West Africa. This lower association of clustering is consistent with investigations from Ghana and The Gambia, which found *M. africanum* less likely to be in spoligotype-defined clusters (*30*,*36*).

After controlling for other factors, we found that TB cases in the United States caused by *M. africanum* and *M. tuberculosis* were similar regarding clinical presentation, social risk factors, and treatment outcomes. These findings are consistent with those of studies that compared treatment outcomes among cases of *M. africanum* and *M. tuberculosis* TB in West Africa, but contrast with studies describing differential associations with HIV and chest radiography findings (*8*,*14*,*28*,*29*). Unlike several reported studies, we could not compare specific chest radiographic findings for *M. africanum* versus *M. tuberculosis* because detailed radiographic information is not available in US surveillance data (*8*). Our study demonstrated similar clinical characteristics of TB caused by L5 and L6 lineages of *M. africanum*, which is consistent with that of a previous report (*29*).

Our results should be interpreted in light of the incomplete availability of genotype data. Nationwide coverage of genotyping has increased over time (*37*), but genotype data were not available for all culture confirmed cases. Although it is possible that our study underestimates the true burden of *M. africanum*, we expect that changes in system coverage do not substantially affect the main findings of the study. In addition, *M. africanum* and *M. tuberculosis* were identified by spoligotype and MIRU-VNTR, rather than by more phylogenetically robust methods, such as large-sequence polymorphism analysis. Therefore, some misclassification might have occurred, but there is no reason to assume any bias was introduced. Finally, our definition of clustered cases was based solely on identical spoligotype and 24-locus MIRU-VNTR in the same county during 2009–2013 and therefore probably overestimates the extent of transmission that might be occurring at the county level. More robust methods for identifying clustered cases rely on a narrower time interval between cases and evidence of epidemiologic links between cases (*38*). Even with the direction of bias toward overestimation of clustering, we found only 1 cluster.

Although the annual number of reported TB cases in the United States has decreased in the past decade, the proportion of TB contributed by foreign-born persons has increased to >60% in recent years (*39*). Similar to this trend, TB caused by *M. africanum* is highest among foreign-born persons, which is consistent with the understanding that spread of *M. africanum* in countries outside Africa is driven by human migration from West Africa. Given the low burden of TB caused by *M. africanum* in the United States, the similarity in clinical features of TB caused by *M. africanum* and *M. tuberculosis*, and the lower odds of clustered cases of *M. africanum* than those of *M. tuberculosis*, routine reporting of TB caused by *M. africanum* above standard reporting for general TB does not appear warranted at this time.

**Technical Appendix.** Characteristics of tuberculosis caused by *Mycobacterium africanum* and *M. tuberculosis* and unique spoligotype and 12-locus and 24-locus mycobacterial interspersed repetitive unit variable number tandem repeat combinations with corresponding number of cases of TB caused by *Mycobacterium africanum*, United States, 2004–2013.
