# Supplementary material for: Tuberculosis Caused by Mycobacterium africanum, United States, 2004–2013
Source: Emerg Infect Dis. 2016 Mar;22(3):396–403. doi: 10.3201/eid2203.151505 (PMC4766873; doi:10.3201/eid2203.151505)
Supplement: Supplementary file 1 — Technical Appendix. Characteristics of tuberculosis caused by Mycobacterium africanum and M. tuberculosis and unique spoligotype and 12-locus and 24-locus mycobacterial interspersed repetitive unit variable number tandem repeat combinations with corresponding number of cases of TB caused by Mycobacterium africanum, United States, 2004–2013. [file 15-1505-Techapp-s1.pdf]

Article DOI: http://dx.doi.org/10.32301.151505

# Tuberculosis Caused by *Mycobacterium africanum*, United States, 2004–2013

## Technical Appendix

**Technical Appendix Table 1.** Characteristics of patients with tuberculosis caused by *Mycobacterium africanum* and *M. tuberculosis*, United States, 2004–2013*

| Variable | No. (%) *M. africanum*, n = 315 | No. (%) *M. tuberculosis*, n = 71,727 | OR (95% CI) | p-value† |
|---|---|---|---|---|
| Country of birth | | | | |
| United States | 39 (12.4) | 28,955 (40.4) | Referent | <0.001 |
| Other | 276 (87.6) | 42,633 (59.6) | 4.8 (3.4–6.7) | |
| Country of origin | | | | |
| In West Africa‡ | 254 (80.6) | 926 (1.3) | 318.4 (239.0–424.2) | <0.001 |
| Not in West Africa | 61 (19.4) | 70,801 (98.7) | Referent | |
| Time in the United States, y§ | | | | |
| 0–2 | 102 (39.5) | 8,998 (23.4) | Referent | <0.001 |
| 2–5 | 64 (24.8) | 6,106 (15.9) | 0.9 (0.7–1.3) | |
| ≥5 | 92 (35.7) | 23,282 (60.7) | 0.3 (0.3–0.5) | |
| Clustered case¶ | | | | |
| Yes | 2 (1.4) | 9,655 (29.0) | 0.1 (0.1–0.2) | <0.001 |
| No | 139 (98.6) | 26,762 (71.0) | Referent | |
| Race/ethnicity | | | | |
| Non–Hispanic White, Asian, or other race | 20 (6.4) | 34,047 (47.6) | Referent | <0.001 |
| Non–Hispanic Black, or multiracial | 286 (91.7) | 18,052 (25.2) | 27.0 (17.1–42.5) | |
| Hispanic | 6 (1.9) | 19,443 (27.2) | 0.5 (0.2–1.3) | |
| Age, y | | | | |
| 0–14 | 7 (2.2) | 1,349 (1.9) | Referent | <0.001 |
| 15–24 | 55 (17.5) | 8,283 (11.5) | 1.3 (0.6–2.8) | |
| 25–44 | 167 (53.0) | 24,122 (33.6) | 1.3 (0.6–2.8) | |
| 45–64 | 68 (21.6) | 22,297 (31.1) | 0.6 (0.3–1.3) | |
| ≥65 | 18 (5.7) | | 0.2 (0.1–0.5) | |
| Sex | | | | |
| F | 118 (37.5) | 26,755 (37.3) | 1.0 (0.8–1.3) | 0.957 |
| M | 197 (62.5) | 44,947 (62.7) | Referent | |
| Reported HIV status | | | | |
| Negative | 207 (65.7) | 46,920 (65.4) | Referent | <0.001 |
| Positive | 56 (17.8) | 4,610 (6.4) | 2.8 (2.0–3.7) | |
| Unknown/not determined | 52 (16.5) | 20,197 (28.2) | 0.6 (0.4–0.8) | |
| Previous diagnosis of TB | | | | |
| Yes | 8 (2.7) | 3,266 (4.6) | 0.6 (0.3–1.1) | 0.107 |
| No | 292 (97.3) | 67,361 (95.4) | Referent | |
| Primary disease site | | | | |
| Pulmonary | 198 (62.9) | 53,350 (74.4) | Referent | <0.001 |
| Extrapulmonary | 76 (24.1) | 11,227 (15.7) | 1.8 (1.4–2.4) | |
| Pulmonary and extrapulmonary | 41 (13.0) | 7,117 (9.9) | 1.6 (1.1–2.2) | |
| Primary extrapulmonary site# | | | | |
| Bone | 13 (17.1) | 1,482 (13.2) | Referent | 0.095** |
| Genitourinary | 3 (3.9) | 637 (5.7) | 0.5 (0.2–1.9) | |
| Cervical lymph node | 17 (22.4) | 3,075 (27.4) | 0.6 (0.3–1.3) | |
| Other lymph node | 14 (18.4) | 1,659 (14.8) | 1.0 (0.5–2.1) | |
| Meningeal | 3 (3.9) | 411 (3.7) | 0.8 (0.2–2.9) | |
| Peritoneal | 6 (7.9) | 661 (5.9) | 1.0 (0.4–2.7) | |
| Pleural | 5 (7.9) | 1,863 (16.6) | 0.3 (0.1–0.9) | |
| Other | 15 (19.7) | 1,439 (12.8) | 1.2 (0.6–2.5) | |
| Chest radiography finding | | | | |
| Abnormal, cavitary | 83 (26.3) | 19,249 (26.8) | 0.6 (0.5–0.9) | <0.001 |
| Abnormal, non-cavitary | 158 (50.0) | 41,794 (58.3) | 0.5 (0.4–0.7) | |
| Normal | 74 (23.7) | 10,684 (14.9) | Referent | |
| Sputum smear result | | | | |
| Positive | 137 (50.7) | 35,063 (56.4) | 0.8 (0.6–1.0) | 0.061 |
| Negative | 133 (49.3) | 27,101 (43.6) | Referent | |

| Variable | No. (%) | | OR (95% CI) | p-value† |
|---|---|---|---|---|
| | *M. africanum*, n = 315 | *M. tuberculosis*, n = 71,727 | | |
| Tuberculin skin test result | | | | |
| Positive | 163 (80.3) | 36,640 (79.8) | 1.0 (0.7–1.5) | 0.853 |
| Negative | 40 (19.7) | 9,292 (20.2) | Referent | |
| Homeless in year before diagnosis | | | | |
| Yes | 8 (2.6) | 4,561 (6.4) | 0.4 (0.2–0.8) | 0.006 |
| No | 304 (97.4) | 66,677 (93.6) | Referent | |
| Resident of correction institution in year before diagnosis | | | | |
| Yes | 2 (0.6) | 2,718 (3.8) | 0.2 (0.0–0.6) | 0.003** |
| No | 313 (99.4) | 68,796 (96.2) | Referent | |
| Resident of long-term care facility in year before diagnosis | | | | |
| Yes | 8 (2.5) | 1,600 (2.2) | 1.1 (0.6–2.3) | 0.716 |
| No | 307 (97.5) | 69,968 (97.8) | Referent | |
| Drug use | | | | |
| Yes | 6 (1.9) | 6,193 (8.8) | 0.2 (0.1–0.5) | <0.001 |
| No | 303 (98.1) | 64,181 (91.2) | Referent | |
| Excessive alcohol use | | | | |
| Yes | 11 (3.5) | 10,135 (14.4) | 0.2 (0.1–0.4) | <0.001 |
| No | 300 (96.5) | 60,383 (85.6) | Referent | |
| Multidrug resistance†† | | | | |
| Yes | 2 (0.6) | 1,006 (1.4) | 0.5 (0.1–1.8) | 0.337** |
| No | 307 (99.4) | 69,847 (98.6) | Referent | |
| Reason therapy stopped | | | | |
| Completed treatment | 271 (91.2) | 57,230 (86.7) | Referent | 0.005 |
| Died during treatment | 8 (2.7) | 5,082 (7.7) | 0.3 (0.2–0.7) | |
| Other reason | 18 (6.1) | 3,668 (5.6) | 1.0 (0.6–1.7) | |
| Reported resistance to isoniazid†† | | | | |
| Resistant | 25 (8.1) | 6,269 (8.8) | 0.9 (0.6–1.4) | 0.644 |
| Susceptible | 284 (91.9) | 64,658 (91.2) | Referent | |
| Reported resistance to rifampin†† | | | | |
| Resistant | 2 (0.6) | 1,235 (1.7) | 0.4 (0.1–1.5) | 0.187** |
| Susceptible | 308 (99.4) | 69,688 (98.3) | Referent | |

*Sums of counts for a variable might be less than the total counts in column headings because of missing data. OR, odds ratio.
†Determined by Pearson $\chi^2$ test unless otherwise specified.
‡Includes Nigeria, Liberia, Sierra Leone, Guinea, The Gambia, Ghana, Mali, Senegal, Côte d'Ivoire, Togo, Cameroon, Mauritania, Niger, and Guinea-Bissau.
§Restricted to cases in foreign-born persons.
¶Restricted to cases reported after 2009, for which complete 24-locus MIRU-VNTR data were available.
#Restricted to cases with only extrapulmonary disease.
**Determined by Fisher exact test.
††Reported resistance is based on results from conventional drug susceptibility testing of the initial specimen on which drug susceptibility testing was performed.

**Technical Appendix Table 2.** Unique spoligotype and 12-locus MIRU-VNTR combinations with corresponding number of cases of TB caused by *Mycobacterium africanum*, United States, 2004–2013*

| Spoligotype | MIRU-VNTR† | No. |
|---|---|---|
| 1111110001111111111111111111111101111101111 | 236214333522 | 13 |
| 1111110001111111111111111111111111111101111 | 226224243521 | 13 |
| 1111110001111111111111111111111111111101111 | 236224233422 | 13 |
| 1111110001111111111110001111111111111101111 | 225424243522 | 9 |
| 1111110000000000111100001111111111110001111 | 224424243221 | 8 |
| 1111111000001111111100001111111111110001111 | 224424244221 | 8 |
| 1111110001111111111110001111111111111101111 | 224424243522 | 7 |
| 1011111000001111111100001111111111110001111 | 224424243221 | 6 |
| 1111111000001111111100001111111111110001111 | 224424243221 | 6 |
| 1111110001111111111111111111111111111101111 | 227224243521 | 5 |
| 1111111000001111111100000000000000000000111 | 224424244221 | 5 |
| 1111111000001111111100001101111101110001111 | 224424244221 | 5 |
| 1111110000000000111100001100111111110001111 | 224424243221 | 4 |
| 1111110001111111111111111111111111111101111 | 225424243522 | 4 |
| 1111111000001111111111111111111111110001111 | 224424244221 | 4 |
| 1001110001111111111111111111111101111101111 | 236214233522 | 3 |
| 1111110001111111111110001111111111111100111 | 225124241522 | 3 |
| 1111110001111111111111111110111101111101111 | 236214333522 | 3 |
| 1111110001111111111111111111111101111100011 | 236214323522 | 3 |
| 1111111000000000000000111111111111110001111 | 124424244221 | 3 |
| 1111111000001111111000001111111111110001111 | 224424234221 | 3 |
| 1111111000001111111100001111110000000001111 | 224424243221 | 3 |
| 1111111000001111111100001111111111110001111 | 224324244221 | 3 |
| 1001111000001111111100001111111111110001111 | 222424243221 | 2 |

| Spoligotype | MIRU-VNTR† | No. |
|---|---|---|
| 1011111000001111111000011111111111110001111 | 224414243221 | 2 |
| 1110000000000000000000000000000000000001111 | 223424243221 | 2 |
| 1111000000000111111111111111111111111101111 | 225424243522 | 2 |
| 1111110000000000011100000000000000000110001111 | 224424243221 | 2 |
| 1111110000000111111110000010000000000001111 | 222324243221 | 2 |
| 1111110001111111111101000111111111111101111 | 224424243522 | 2 |
| 1111110001111111111110001111111111111101111 | 224324243522 | 2 |
| 1111110001111111111111111111111111111101111 | 226224253521 | 2 |
| 1111110001111111111111111111111111111101111 | 227224243421 | 2 |
| 1111110001111111111111111111111111111101111 | 235224233422 | 2 |
| 1111111000000000111111111111111111010001111 | 224424242221 | 2 |
| 1111111000000011110000001111111111110001111 | 224424234221 | 2 |
| 1111111000001011111100001111111111110001111 | 224324243221 | 2 |
| 1111111000001111011000000111111000010001111 | 224224244221 | 2 |
| 1111111000001111110000001111111111110001111 | 224424244221 | 2 |
| 1111111000001111111000000000000000000001111 | 224424244221 | 2 |
| 1111111000001111111000001000000000000000111 | 224424244221 | 2 |
| 1111111000001111111000011111111111110000111 | 224424254221 | 2 |
| 1111111000001111111000011111111111110001101 | 224424244221 | 2 |
| 1111111000001111111000011111111111110001111 | 222424244221 | 2 |
| 0111111000001111111000011111111111110000011 | 224424244221 | 1 |
| 0111111000001111111000011111111111110000111 | 224324244221 | 1 |
| 0111111000001111111000011111111111110000111 | 224424–24221 | 1 |
| 1010111000001111111000011111111111110001111 | 224424243221 | 1 |
| 1011011000001001111100001111111011110001101 | 224424244221 | 1 |
| 1011110000000000111100001111111111110001111 | 224424243221 | 1 |
| 1011111000001111111000011111111111110000111 | 2244–4243221 | 1 |
| 1011111000001111111000011111111111110001111 | 224424143221 | 1 |
| 1011111000001111111111111111111111110001111 | 224425244221 | 1 |
| 1101111000000111111111111111111111110000111 | 224424244221 | 1 |
| 1101111000001111111100111111111111110001111 | 234424224221 | 1 |
| 1110000000000000000111111111111111101101111 | 237224243511 | 1 |
| 1110000000001111011111111111100000001101111 | 224325253324 | 1 |
| 1110000000001111111111111111111111111101111 | 126224243521 | 1 |
| 1110000000111111100011111111111111111101111 | 236224233412 | 1 |
| 1110000000111111100011111111111111111101111 | 236224233422 | 1 |
| 1110010001111111111111111111111111111101111 | 226224243521 | 1 |
| 1110011000001111111111101000000000110001111 | 224424244221 | 1 |
| 1110111000001111111000011111111111110001111 | 224424243221 | 1 |
| 1110111000001111111111111111111111110001111 | 224324244221 | 1 |
| 1111011000001111111000000000000000010001111 | 224424244221 | 1 |
| 1111100000000000000111111111111111111101111 | 226224243521 | 1 |
| 1111100000111111101111111111111000111101111 | 225224243521 | 1 |
| 1111110000000000010110000111111111110001111 | 224424243221 | 1 |
| 1111110000000000011100000000000000000110001110 | 223424243221 | 1 |
| 1111110000000000011100000000000000000110001111 | 224524243221 | 1 |
| 1111110000000000011110000111111111110000001 | 234424243221 | 1 |
| 1111110000000111111100000000000000000001111 | 222324243221 | 1 |
| 1111110000000111111100000000000000000001111 | 222424243221 | 1 |
| 1111110000111001111111111111111110111110110 1 | 227224243521 | 1 |
| 1111110001001111111111111111111111111101111 | 227224243321 | 1 |
| 1111110001100111111111111111111011111101111 | 226214333522 | 1 |
| 1111110001100111111111111111111011111101111 | 236214333822 | 1 |
| 1111110001110111110000001000011111111101111 | 213424243522 | 1 |
| 1111110001110111111110001111111111111101111 | 224424243522 | 1 |
| 1111110001110111111111111111111111111101111 | 225424243522 | 1 |
| 1111110001110111111111111111111111111101111 | 226224233422 | 1 |
| 1111110001110111111111111111111111111101111 | 227224243521 | 1 |
| 1111110001111000000000110111111111111001111 | 227224243521 | 1 |
| 1111110001111110000001111111111111111101111 | 227224243521 | 1 |
| 1111110001111111110000001000011111110101111 | 214324231522 | 1 |
| 1111110001111111110001111111111111111101111 | 226224233521 | 1 |
| 1111110001111111111011111111111111111101111 | 226224243521 | 1 |
| 1111110001111111111110000111111011111101111 | 225424–33522 | 1 |
| 1111110001111111111110001110001111111101011 | 225424243522 | 1 |
| 1111110001111111111110001111111101111100111 | 225124241522 | 1 |
| 1111110001111111111110001111111110110101111 | 225424243522 | 1 |
| 1111110001111111111110001111111111111100111 | 225424243522 | 1 |
| 1111110001111111111110001111111111111101011 | 224424243522 | 1 |
| 1111110001111111111110001111111111111101100 | 225424243522 | 1 |
| 1111110001111111111110001111111111111101111 | 213424273522 | 1 |
| 1111110001111111111110001111111111111101111 | 215424243522 | 1 |
| 1111110001111111111110001111111111111101111 | 224424243722 | 1 |

| Spoligotype | MIRU-VNTR† | No. |
|---|---|---|
| 1111110001111111111110001111111111111101111 | 224624243522 | 1 |
| 1111110001111111111110001111111111111101111 | 225324243422 | 1 |
| 1111110001111111111110001111111111111101111 | 225324243522 | 1 |
| 1111110001111111111110001111111111111101111 | 225424233522 | 1 |
| 1111110001111111111110001111111111111101111 | 225424243622 | 1 |
| 1111110001111111111110001111111111111101111 | 225425243522 | 1 |
| 1111110001111111111111111111111000001101111 | 226224243521 | 1 |
| 1111110001111111111111111111111000011101111 | 236214233422 | 1 |
| 1111110001111111111111111111111101111100011 | 226214333522 | 1 |
| 1111110001111111111111111111111101111100011 | 236214323422 | 1 |
| 1111110001111111111111111111111101111100011 | 236214333522 | 1 |
| 1111110001111111111111111111111101111101111 | 235214233523 | 1 |
| 1111110001111111111111111111111101111101111 | 236212333522 | 1 |
| 1111110001111111111111111111111101111101111 | 236214233521 | 1 |
| 1111110001111111111111111111111101111101111 | 236214333523 | 1 |
| 1111110001111111111111111111111111111100111 | 222224243521 | 1 |
| 1111110001111111111111111111111111111100111 | 226224243521 | 1 |
| 1111110001111111111111111111111111111100111 | 227224243521 | 1 |
| 1111110001111111111111111111111111111100111 | 236224233422 | 1 |
| 1111110001111111111111111111111111111101101 | 226224243521 | 1 |
| 1111110001111111111111111111111111111101101 | 236224233422 | 1 |
| 1111110001111111111111111111111111111101110 | 215224233522 | 1 |
| 1111110001111111111111111111111111111101110 | 236224233422 | 1 |
| 1111110001111111111111111111111111111101111 | 127224243321 | 1 |
| 1111110001111111111111111111111111111101111 | 2–5424243522 | 1 |
| 1111110001111111111111111111111111111101111 | 223224243521 | 1 |
| 1111110001111111111111111111111111111101111 | 225224243521 | 1 |
| 1111110001111111111111111111111111111101111 | 226224233521 | 1 |
| 1111110001111111111111111111111111111101111 | 226224243–21 | 1 |
| 1111110001111111111111111111111111111101111 | 226224243511 | 1 |
| 1111110001111111111111111111111111111101111 | 226224243721 | 1 |
| 1111110001111111111111111111111111111101111 | 227224213521 | 1 |
| 1111110001111111111111111111111111111101111 | 227224242321 | 1 |
| 1111110001111111111111111111111111111101111 | 227224243321 | 1 |
| 1111110001111111111111111111111111111101111 | 227224263521 | 1 |
| 1111110001111111111111111111111111111101111 | 23–224233422 | 1 |
| 1111110001111111111111111111111111111101111 | 2352–4232422 | 1 |
| 1111110001111111111111111111111111111101111 | 237224233422 | 1 |
| 1111110001111111111111111111111111111101111 | 238224233422 | 1 |
| 1111110001111111111111111111111111111101111 | 325224243521 | 1 |
| 1111111000000000000000000000000000000000001 | 224424243221 | 1 |
| 1111111000000000000000000000000000010001111 | 224524244221 | 1 |
| 1111111000000000000000111111111111111100111 | 124424244221 | 1 |
| 1111111000000011111100001111111111110000111 | 234416244221 | 1 |
| 1111111000000011111100001111111111110001111 | 224624243221 | 1 |
| 1111111000000011111111111111111111110000111 | 224424244221 | 1 |
| 1111111000000011111111111111111111110001111 | 224424244221 | 1 |
| 1111111000000111111100001111111111110001111 | 224424243221 | 1 |
| 1111111000001011111111111111111111100001111 | 224424254221 | 1 |
| 1111111000001100001111110000000000100001111 | 224424244221 | 1 |
| 1111111000001101111111111111111111110001111 | 224424253221 | 1 |
| 1111111000001111110000001011111111110001111 | 224224244221 | 1 |
| 1111111000001111111000000001100000110000111 | 224424244221 | 1 |
| 1111111000001111111000000101100000110000111 | 225424244221 | 1 |
| 1111111000001111111000000101110001110000111 | 224424244231 | 1 |
| 1111111000001111111000001111111111110001111 | 224524244221 | 1 |
| 1111111000001111111100000000000000000000111 | 224524244221 | 1 |
| 1111111000001111111100000100000000010001111 | 224424244221 | 1 |
| 1111111000001111111100001111101111110001111 | 224424234221 | 1 |
| 1111111000001111111100001111111000000001111 | 224324243221 | 1 |
| 1111111000001111111100001111111100010000111 | 224414244221 | 1 |
| 1111111000001111111100001111111111100001111 | 224424243221 | 1 |
| 1111111000001111111100001111111111110000111 | 224324244221 | 1 |
| 1111111000001111111100001111111111110000111 | 224414–44221 | 1 |
| 1111111000001111111100001111111111110000111 | 224414244221 | 1 |
| 1111111000001111111100001111111111110001001 | 223424234221 | 1 |
| 1111111000001111111100001111111111110001111 | 214424243221 | 1 |
| 1111111000001111111100001111111111110001111 | 223424354221 | 1 |
| 1111111000001111111100001111111111110001111 | 224324213221 | 1 |
| 1111111000001111111100001111111111110001111 | 224324243221 | 1 |
| 1111111000001111111100001111111111110001111 | 224325244221 | 1 |
| 1111111000001111111100001111111111110001111 | 224414243221 | 1 |
| 1111111000001111111100001111111111110001111 | 224424143221 | 1 |

| Spoligotype | MIRU-VNTR† | No. |
|---|---|---|
| 1111111000001111111100001111111111110001111 | 224424211221 | 1 |
| 1111111000001111111100001111111111110001111 | 225424243221 | 1 |
| 1111111000001111111101100000000000010001111 | 224624244221 | 1 |
| 1111111000001111111111100000000001010001111 | 234424244221 | 1 |
| 1111111000001111111111101000000000010001111 | 224614244221 | 1 |
| 1111111000001111111111111111111111010001111 | 224424242221 | 1 |
| 1111111000001111111111111111111111110001111 | 223424244221 | 1 |
| 1111111000001111111111111111111111110001111 | 224224244221 | 1 |
| 1111111000001111111111111111111111110001111 | 224324244221 | 1 |
| 1111111000001111111111111111111111110001111 | 224424234221 | 1 |
| 1111111000001111111111111111111111110001111 | 224424242221 | 1 |
| 1111111000001111111111111111111111110001111 | 224424245221 | 1 |
| 1111111000001111111111111111111111110001111 | 224524244221 | 1 |

*MIRU-VNTR, mycobacterial interspersed repetitive unit variable number tandem repeat.
†Digits refer to number of repeats detected at the respective 12 MIRU-VNTR locus in the following order: 2, 4 (ETR D), 10, 16, 20, 23, 24, 26, 27 (QUB-5), 31 (ETR E), 39, 40.

**Technical Appendix Table 3.** Unique spoligotype and 24-locus MIRU-VNTR combinations with corresponding number of cases of tuberculosis caused by *Mycobacterium africanum*, United States, 2009–2013*

| Spoligotype | MIRU-VNTR† | No. |
|---|---|---|
| 1111110001111111111111111111111111111101111 | 236224233422 253463443434 | 7 |
| 1011111000001111111100001111111111110001111 | 224424243221 142–43423332 | 3 |
| 1111110000000000111100001111111111110001111 | 224424243221 142–43423332 | 3 |
| 1111111000001111111100001111111111110001111 | 224424244221 342–43423232 | 3 |
| 1110000000000000000000000000000000000001111 | 223424243221 142–43223332 | 2 |
| 1111110001111111111110001111111111111101111 | 225424243522 354563443316 | 2 |
| 1111111000001111111100001101111101110001111 | 224424244221 142–43423332 | 2 |
| 1111111000001111111100001111111111110001111 | 222424244221 343–43423332 | 2 |
| 1111111000001111111100001111111111110001111 | 224324244221 142–43423332 | 2 |
| 1111111000001111111100001111111111110001111 | 224424243221 142–43423332 | 2 |
| 0111111000001111111100001111111111110000011 | 224424244221 342–43433333 | 1 |
| 0111111000001111111100001111111111110000111 | 224324244221 342–43423333 | 1 |
| 1001110001111111111111111111111101111101111 | 236214233522 153473443436 | 1 |
| 1001110001111111111111111111111101111101111 | 236214233522 253573443436 | 1 |
| 1010111000001111111100001111111111110001111 | 224424243221 122–43423332 | 1 |
| 1011011000001001111100001111111011110001101 | 224424244221 342–43423212 | 1 |
| 1011111000001111111100001111111111110000111 | 2244–4243221 142643423432 | 1 |
| 1011111000001111111100001111111111110001111 | 224414243221 142–43423332 | 1 |
| 1011111000001111111111111111111111110001111 | 224425244221 442–43424333 | 1 |
| 1101111000000111111111111111111111110000111 | 224424244221 342–33423433 | 1 |
| 1110000000000000000011111111111111101101111 | 237224243511 253463432636 | 1 |
| 1110000000111111110001111111111111111101111 | 236224233422 253463443434 | 1 |
| 1110010001111111111111111111111111111101111 | 226224243521 254443453436 | 1 |
| 1110011000001111111111101000000000110001111 | 224424244221 342–33423333 | 1 |
| 1110111000001111111111111111111111110001111 | 224324244221 342–43523333 | 1 |
| 1111011000001111111100000000000000010001111 | 224424244221 342–43423233 | 1 |
| 1111110000000000111000000000000000110001110 | 223424243221 142–43423332 | 1 |
| 1111110000000000111100001100111111110001111 | 224424243221 122–43423232 | 1 |
| 1111110000000000111100001100111111110001111 | 224424243221 122243423232 | 1 |
| 1111110000000000111100001100111111110001111 | 224424243221 142–43423331 | 1 |
| 1111110000000000111100001100111111110001111 | 224424243221 142–43423332 | 1 |
| 1111110000000000111100001111111111110000001 | 234424243221 142–33423332 | 1 |
| 1111110000000000111100001111111111110001111 | 224424243221 142–43423432 | 1 |
| 1111110000000000111100001111111111110001111 | 224424243221 142233423332 | 1 |
| 1111110001001111111111111111111111111101111 | 227224243321 264473443–36 | 1 |
| 1111110001110111110000001000011111111101111 | 213424243522 354463443436 | 1 |
| 1111110001110111111110001111111111111101111 | 224424243522 254563453436 | 1 |
| 1111110001110111111111111111111111111101111 | 226224233422 253463443434 | 1 |
| 1111110001111111000000111111111111111101111 | 227224243521 254483443436 | 1 |
| 1111110001111111111101111111111111111101111 | 226224243521 254473433436 | 1 |
| 1111110001111111111110001111111110110101111 | 225424243522 354563443236 | 1 |
| 1111110001111111111110001111111111111100111 | 225424243522 354563442436 | 1 |
| 1111110001111111111110001111111111111101100 | 225424243522 354563443436 | 1 |
| 1111110001111111111110001111111111111101111 | 215424243522 356363443436 | 1 |
| 1111110001111111111110001111111111111101111 | 224324243522 354363443433 | 1 |
| 1111110001111111111110001111111111111101111 | 224424243522 3545–3443436 | 1 |
| 1111110001111111111110001111111111111101111 | 224424243522 354563443436 | 1 |
| 1111110001111111111110001111111111111101111 | 225324243522 354563443436 | 1 |
| 1111110001111111111110001111111111111101111 | 225424243522 354563433436 | 1 |
| 1111110001111111111110001111111111111101111 | 225424243522 366563443436 | 1 |

| Spoligotype | MIRU-VNTR† | No. |
|---|---|---|
| 1111110001111111111110001111111111111101111 | 225424243622 354563433436 | 1 |
| 1111110001111111111111111110111011111101111 | 236214333522 253443443436 | 1 |
| 1111110001111111111111111111111101111100011 | 236214323422 253473443436 | 1 |
| 1111110001111111111111111111111101111100011 | 236214323522 253473443433 | 1 |
| 1111110001111111111111111111111101111100011 | 236214323522 253473443436 | 1 |
| 1111110001111111111111111111111101111100011 | 236214333522 253473443436 | 1 |
| 1111110001111111111111111111111101111101111 | 235214233523 253473443436 | 1 |
| 1111110001111111111111111111111101111101111 | 236212333522 253473443436 | 1 |
| 1111110001111111111111111111111101111101111 | 236214333522 253–43443436 | 1 |
| 1111110001111111111111111111111101111101111 | 236214333522 253–73443436 | 1 |
| 1111110001111111111111111111111101111101111 | 236214333522 253363443436 | 1 |
| 1111110001111111111111111111111101111101111 | 236214333522 253363444435 | 1 |
| 1111110001111111111111111111111101111101111 | 236214333522 253443443436 | 1 |
| 1111110001111111111111111111111101111101111 | 236214333522 253473443%36 | 1 |
| 1111110001111111111111111111111101111101111 | 236214333522 254443443436 | 1 |
| 1111110001111111111111111111111111111100111 | 222224243521 254373453436 | 1 |
| 1111110001111111111111111111111111111100111 | 236224233422 252463443434 | 1 |
| 1111110001111111111111111111111111111101111 | 127224243321 254473443436 | 1 |
| 1111110001111111111111111111111111111101111 | 223224243521 254473453436 | 1 |
| 1111110001111111111111111111111111111101111 | 225224243521 354473453426 | 1 |
| 1111110001111111111111111111111111111101111 | 226224233521 253533443436 | 1 |
| 1111110001111111111111111111111111111101111 | 226224243–21 254473453436 | 1 |
| 1111110001111111111111111111111111111101111 | 226224243511 254473453226 | 1 |
| 1111110001111111111111111111111111111101111 | 226224243521 254373453436 | 1 |
| 1111110001111111111111111111111111111101111 | 226224243521 254443443436 | 1 |
| 1111110001111111111111111111111111111101111 | 226224243521 254473343436 | 1 |
| 1111110001111111111111111111111111111101111 | 226224243521 254473453434 | 1 |
| 1111110001111111111111111111111111111101111 | 226224243521 254473453634 | 1 |
| 1111110001111111111111111111111111111101111 | 226224243521 264373453436 | 1 |
| 1111110001111111111111111111111111111101111 | 227224213521 254473443634 | 1 |
| 1111110001111111111111111111111111111101111 | 227224243321 254373443436 | 1 |
| 1111110001111111111111111111111111111101111 | 227224243421 254473453436 | 1 |
| 1111110001111111111111111111111111111101111 | 227224243521 254473443434 | 1 |
| 1111110001111111111111111111111111111101111 | 227224243521 254473443534 | 1 |
| 1111110001111111111111111111111111111101111 | 2352–4232422 2–34–3443434 | 1 |
| 1111110001111111111111111111111111111101111 | 235224233422 253463443434 | 1 |
| 1111110001111111111111111111111111111101111 | 237224233422 253463443434 | 1 |
| 1111111000000000000000000000000000010001111 | 224524244221 342–43413333 | 1 |
| 1111111000000000000000111111111111110001111 | 124424244221 142–43423333 | 1 |
| 1111111000000011111100001111111111110001111 | 224624243221 142–43423332 | 1 |
| 1111111000000011111111111111111111110000111 | 224424244221 342–33423433 | 1 |
| 1111111000001011111100001111111111110001111 | 224324243221 342–43423332 | 1 |
| 1111111000001011111100001111111111110001111 | 224324243221 442–43223333 | 1 |
| 1111111000001100001111110000000001100001111 | 224424244221 342–43423332 | 1 |
| 1111111000001111011000000111111000010001111 | 224224244221 342–43423332 | 1 |
| 1111111000001111111000000111111111110001111 | 224424244221 242–43423313 | 1 |
| 1111111000001111111100000000000000000000111 | 224424244221%42–43423533 | 1 |
| 1111111000001111111100000000000000000000111 | 224424244221 242–43423533 | 1 |
| 1111111000001111111100000000000000000001111 | 224424244221 342423423332 | 1 |
| 1111111000001111111100001111101111110001111 | 224424234221 242–43423332 | 1 |
| 1111111000001111111100001111111000000001111 | 224324243221 142–43423332 | 1 |
| 1111111000001111111100001111111000000001111 | 224424243221 142–43423632 | 1 |
| 1111111000001111111100001111111100010000111 | 224414244221 342–43423333 | 1 |
| 1111111000001111111100001111111111100001111 | 224424243221 142243423332 | 1 |
| 1111111000001111111100001111111111110000111 | 224424254221 242243423333 | 1 |
| 1111111000001111111100001111111111110001101 | 224424244221 3—43423332 | 1 |
| 1111111000001111111100001111111111110001111 | 223424354221 342–43423232 | 1 |
| 1111111000001111111100001111111111110001111 | 224324244221 342–43423232 | 1 |
| 1111111000001111111100001111111111110001111 | 224325244221 342–23422432 | 1 |
| 1111111000001111111100001111111111110001111 | 224414243221 152–43423332 | 1 |
| 1111111000001111111100001111111111110001111 | 224424143221 142–43433332 | 1 |
| 1111111000001111111100001111111111110001111 | 224424243221 142–33423332 | 1 |
| 1111111000001111111100001111111111110001111 | 224424243221 142–43422332 | 1 |
| 1111111000001111111100001111111111110001111 | 224424243221 142–43423232 | 1 |
| 1111111000001111111100001111111111110001111 | 224424244221 242–43423232 | 1 |
| 1111111000001111111100001111111111110001111 | 224424244221 343–43423332 | 1 |
| 1111111000001111111111110000000001010001111 | 234424244221 342–43223333 | 1 |
| 1111111000001111111111101000000000010001111 | 224614244221 342–43421433 | 1 |
| 1111111000001111111111111111111111010001111 | 224424242221 342–43423533 | 1 |
| 1111111000001111111111111111111111110001111 | 223424244221 342–43423333 | 1 |
| 1111111000001111111111111111111111110001111 | 224424244221 3—-3423333 | 1 |
| 1111111000001111111111111111111111110001111 | 224424244221 342–43423333 | 1 |
| 1111111000001111111111111111111111110001111 | 224424244221 342233423333 | 1 |

| Spoligotype | MIRU-VNTR† | No. |
| --- | --- | --- |

*MIRU-VNTR, mycobacterial interspersed repetitive unit variable number tandem repeat.

†Digits refer to number of repeats detected at the respective 24 MIRU-VNTR locus in the following order: 2, 4 (ETR D), 10, 16, 20, 23, 24, 26, 27 (QUB-5), 31 (ETR E), 39, 40, 424 (Mtub04), 577 (ETR C), 1955 (Mtub21), 2163b (QUB-11b), 2165 (ETR A), 2347 (Mtub 29), 2401 (Mtub 3), 2461 (ETR B), 3171 (Mtub 24), 3690 (Mtub 39), 4156 (QUB-4156), 4052 (QUB-26).
